# Building AI competence in the healthcare workforce with the AI for clinical care workshop: A Bridge2AI for clinical CHoRUS project

**DOI:** 10.1017/cts.2025.10156

**Published:** 2025-10-03

**Authors:** Andrea E. Davidson, Aiden Jose, Benjamin Shickel, Kaleb E. Smith, Parisa Rashidi, Yulia Levites Strekalova, Azra Bihorac

**Affiliations:** 1 Intelligent Clinical Care Center, University of Florida, Gainesville, FL, USA; 2 Division of Nephrology, Hypertension, and Renal Transplantation, Department of Medicine, University of Florida, Gainesville, FL, USA; 3 NVIDIA, Santa Clara, CA, USA; 4 J. Crayton Pruitt Family Department of Biomedical Engineering, University of Florida, Gainesville, FL, USA; 5 Department of Health Services Research, Management and Policy, College of Public Health and Health Professions, University of Florida, Gainesville, FL, USA

**Keywords:** Artificial intelligence (AI), AI training, workforce training, workshop development, medical ai training, AI bootcamp, Bridge2AI, CHoRUS

## Abstract

**Background:**

The implementation of artificial intelligence (AI) tools into clinical spheres emphasizes the critical need for an AI-competent healthcare workforce that can interpret AI output and identify its limitations. Without comprehensive training, there is a risk of misapplication, mistrust, and underutilization. Workforce skill development events such as workshops and hackathons can increase AI competence and foster interdisciplinary collaboration to promote optimal patient care.

**Methods:**

The University of Florida hosted the AI for Clinical Care (AICC) workshop in April 2024 to address the need for AI-competent healthcare professionals. The hybrid workshop featured a beginner and advanced track with interactive sessions, hands-on skill development, and networking opportunities led by experts. An anonymous, voluntary post-workshop survey asked participants to score their knowledge and skills before and after the AICC workshop. A second, follow-up survey was administered approximately nine months later.

**Results:**

Ninety participants attended the AICC workshop, forty-one attendees completed the post-workshop survey, and six attendees completed the follow-up survey. Paired *T*-tests of the post-workshop survey revealed statistically significant (*P* < .001) increases in self-reported knowledge gain across all six beginner track learning objectives and significant (*P* < .05) increases across all five advanced track objectives. Feedback indicated participants appreciated the interactive format, although communication and networking needed improvement.

**Conclusion:**

The AICC workshop successfully advanced AI literacy among biomedical professionals and promoted collaborative peer networks. Continued efforts are recommended to enhance participant engagement and ensure equitable access to AI education in clinical settings.

## Introduction

Artificial intelligence (AI) is set to revolutionize scientific fields, particularly biomedicine, where new applications continue to emerge in clinical care. The growing recognition of AI’s powerful capabilities to diagnose patients, predict patient prognosis, interpret medical imagining, and recommend treatment plans suggests that AI and machine learning’s (AI/ML) most significant impacts on people’s lives could arise from their implementation into the clinical domain [1]. As such, there is an increasing demand for medical professionals with competence in these fields who can leverage their understanding of the nuances in clinical data to develop safe and effective medical AI models [2,3]. AI’s potential to transform the way healthcare providers practice medicine also underscores the need for early exposure and training in AI/ML disciplines. Academic institutions must adapt to the changing clinical landscape to equip tomorrow’s doctors with an understanding of the principles and development process of AI/ML assistive devices. Proficiency in these disciplines will promote effective implementation of AI into clinical practices and strengthen the practitioners’ capacity to interpret and contextualize AI-generated results while maintaining an awareness of their limitations.

Medical AI is inherently multidisciplinary, drawing from the biomedical, data, computer, engineering, and translational sciences. Thus, creating opportunities for early-stage investigators, medical and graduate students, and physicians-in-training to engage in interdisciplinary collaboration is essential for developing innovative solutions to address complex clinical challenges and cultivating future leaders in healthcare innovation. To this end, efforts are growing to broaden participation in AI/ML skill development. Initiatives such as hack and data marathons, commonly referred to as hackathons and datathons, have brought interdisciplinary teams together for rapid software prototyping and data analytics projects. Such events, which often involve the collaboration of clinicians and data scientists to analyze datasets to answer a research question, encourage the exchange of knowledge and ideas across disciplines and serve as a networking opportunity for those in attendance. Piza et al. conducted a health datathon event and reported the results of participant surveys, finding that teamwork variables and effective leadership were strongly associated with higher scores of self-reported affective learning [4]. The importance of effective leadership in these skill-development events and the scarcity of clinicians trained in computer science illustrates a need for focused learning events that provide hands-on training and personalized mentoring and promote community building.

The implementation of data-driven technologies in healthcare prompted the need to equip clinicians with AI and machine learning (ML) competencies for successful technology use and implementation in healthcare practices [3]. The implementation of AI/ML in healthcare requires significant resources and skills to navigate them, including data access and technological infrastructure [5]. Furthermore, while AI tools can enhance healthcare delivery, they are often considered “black boxes” by clinicians who are skeptical about trusting them [6]. In recent years, short-duration intensive programs and bootcamps have emerged as effective training approaches for workforce upskilling by providing concentrated exposure to AI training for clinical and biomedical applications. Bootcamps, or day-long workshops, are among the approaches that accommodate the busy schedules of practicing clinicians and provide a conducive environment to learn and practice AI/ML skills. These educational formats offer intensive, focused learning experiences that can significantly enhance clinicians’ understanding and application of AI/ML in their practice. Clinicians often face significant time constraints, making participating in extended training programs challenging. Bootcamps, being concentrated learning experiences that can significantly enhance participants’ skills in a short period, are well-suited to fit into their schedules, providing a viable solution to this barrier [7]. Bootcamps can also effectively engage participants through diverse teaching modalities such as interactive and varied content, individual and group work, and peer feedback to maintain engagement and facilitate deeper learning. Overall, this method holds promise for AI/ML training for clinicians, ensuring that they can apply new skills directly to their work.

The effectiveness of bootcamps depends heavily on immediately applicable skills with focused learning objectives rather than comprehensive technical training. Furthermore, training design should consider and capitalize on the needs and motivations of these accomplished professionals as adult learners [8]. Adult learning theory provides a comprehensive foundation for designing effective AI training programs. Unlike traditional pedagogical approaches focused on passive information absorption, adult learning theory emphasizes active engagement, relevance, and practical application that resonates with learners’ existing professional contexts [9]. Effective AI training must, therefore, emphasize practical applications specific to clinical and biomedical research workflows rather than presenting abstract technical concepts in isolation [5].

We report on the development, implementation, and evaluation of an AI for Clinical Care (AICC) workshop organized by the National Institutes of Health (NIH) Bridge to Artificial Intelligence (Bridge2AI) consortium. Designed as a hybrid, single-day bootcamp for early-career clinicians and established data scientists alike, the AICC workshop offered concurrent beginner and advanced track courses and networking sessions for in-person participants. Following the event, participants were invited to complete a voluntary post-workshop survey to share feedback and score their pre- and post-workshop knowledge and skill levels for each learning objective.

## Methods

### Program background

The second annual AICC workshop took place in April 2024 and was hosted in collaboration with the NIH Bridge2AI program through the Patient-Focused Collaborative Hospital Repository Uniting Standards (CHoRUS) for Equitable AI network. The event was held in collaboration with the University of Florida (UF) College of Medicine, Intelligent Clinical Care Center (IC^3^), Herbert Wertheim College of Engineering, and in partnership with NVIDIA. Workshop structure and content was developed by professionals from the University of Florida and NVIDIA. A complete list of Bridge2AI CHoRUS consortia authors can be found in Supplementary Materials 5.

The NIH Common Fund’s Bridge2AI program is a consortium of institutions involved in biomedical, behavioral, and AI/ML research initiatives [10–12]. The program’s goal is to promote the development of standards, ethics, tools, data sets, and workforce development strategies in the AI scientific community.

The Patient-Focused CHoRUS for Equitable AI is one of four data generation projects in the Bridge2AI consortium. The goal of CHoRUS is to develop national infrastructure for AI research through the development of a high-resolution, diverse, and ethically sourced AI-ready data set from critically ill patients from 14 data acquisition centers across the US [12]. The CHoRUS project will build upon this data set to develop standards and tools to facilitate uniformity between data sourced from different sites and expand the AI in critical care research workforce. This Skills and Workforce Development component of the CHoRUS project, led by investigators at the University of Florida, aims to develop and implement educational activities around CHoRUS data and tools to facilitate skill development in the next generation of researchers in AI in critical care medicine. Moreover, this module aims to diversify the AI research workforce through the development of outreach, educational, and development programs for underrepresented communities.

### Workshop design

The AICC workshop was a hybrid, single-day, hands-on skill development workshop held in April 2024 at the UF Lake Nona Research Facility in Orlando, Florida. Those who attended virtually were invited to join via Zoom video conference. The workshop was tailored to address the skill development needs of those with limited prior experience with AI/ML and those who were interested in advancing their existing AI/ML competencies. The workshop consisted of two tracks: an AI Boot Camp beginner track and an advanced track for Generative AI with Diffusion Models Workshop. The beginner track, taught by CHoRUS and UF College of Medicine AI faculty, was designed for individuals without any prior programming experience and comprised of micro-learning tutorials showcasing AI/ML methods applied to real-world clinical datasets. The advanced track was taught by NVIDIA data scientists and followed the NVIDIA Deep Learning Institute course on Generative AI and denoising diffusion models. The advanced track’s focus on Generative AI, though not directly clinical, was offered to attract experienced AI/ML professionals through a specialized and high-interest topic. By encouraging the participation of AI/ML experts who may work outside of healthcare applications to learn in a shared environment, we aimed to promote interdisciplinary collaboration and networking across clinical and technical domains. Those who attended in person were invited to attend a pre-workshop breakfast, which included a brief interactive networking activity. Following breakfast, participants were split into the morning sessions of either the beginner or advanced workshop track. All participants reconvened for a mid-day networking lunch before breaking off into the afternoon concurrent workshop tracks. Workshop agendas can be found in Supplementary Materials 1.

Following the conclusion of the AICC workshop, an optional post-workshop survey was administered to attendees. This survey was designed to capture feedback regarding attendee knowledge before and after the event. Attendees were also asked to provide feedback on the workshop content, mentoring, and networking sessions.

### Beginner track curriculum development

The AICC beginner track curriculum was developed with four primary goals in mind: (1) demystify AI with hands-on experiential learning, (2) define a *core* AI vocabulary of fundamental concepts and terminology, (3) identify current real-world clinical AI applications, issues, and ethical considerations, and (4) facilitate future self-learning and exploration after the conclusion of the workshop.

Prior to the AICC workshop, beginner track participants were instructed to complete two self-paced training courses offered by the University of Florida Office of Professional and Workforce Development. These included a four-hour *Foundations of AI-Based Medicine* course and an 8-hour *AI-Based Medicine Technical Expertise* course. Registration fees are typically $200 each but were waived for workshop participants.

The workshop was largely conducted using Jupyter notebooks, an interactive Python programming file that allows the interleaving of executable code with text, images, and other multimedia elements helpful for instruction. Each participant worked through an individual version of each of the workshop’s six notebooks on their own laptops. Four of the notebooks (*Introduction to Python, Biomedical Data Analysis, Machine Learning for Clinical Care, Introduction to Deep Learning*) consisted of guided group instruction by an expert instructor, and two (*Introduction to Version Control* and *Clinical AI Ethics*) were self-paced. During all six notebook sessions, questions were encouraged, and discussions were frequent. Three UF PhD students with expertise in AI, ML, and deep learning applications were available as teaching assistants to answer questions from virtual and in-person participants.

The sequence of notebooks and their targeted instructional subtopics were carefully selected so that each participant could end the day with the knowledge and experience of developing and evaluating their own real-world machine learning prediction model on a dataset of electronic health records. Each notebook’s concepts were taught using a cascading approach, where only the information required for the next notebook was covered in the current notebook. The curriculum was largely determined in reverse order from the primary final objective of training a clinical AI model.

Importantly, this workshop did not explore the low-level mathematical foundations of machine learning. While these details are important for a complete understanding of AI, the curriculum instead focused on the application of AI to clinical problems, discussing contextual questions and indications of when, where, how, and why to use AI for a given healthcare application – much like treating AI as clinical tool or medical device.

Following the final Jupyter notebook lesson were three presentations on the AI clinical applications, multi-modal waveform analysis, and the standardization of AI-ready data from healthcare settings. Presentations entitled *Multi-modal Models of Physiological Deterioration and their Implications on Ethical AI* and *Observational Health Data Sciences and Informatics: Open Science Ecosystem and AI Tools* were presented by two CHoRUS Principal Investigators, and *Introduction to Clinical Applications of AI* was presented by a UF Clinical Associate Professor.

### Advanced track curriculum development

The AICC advanced track followed the NVIDIA Deep Learning Institute’s *Generative AI with Diffusion Models* course and was intended for those experienced with deep learning concepts. While this course is available as an online self-paced course, AICC workshop advanced track attendees had the opportunity to learn directly from NVIDIA data scientists leading the course. Unlike the beginner track, those wishing to attend the advanced track workshop were required to complete a questionnaire to determine their eligibility for the course (Supplementary Materials 2). A sufficient understanding of deep learning modeling and the PyTorch framework was required. Questionnaire responses were individually considered by the NVIDIA course instructors, and participants were accepted on a case-by-case basis.

The Deep Learning Institute strives to offer training that is up-to-date with modern AI practices. Given NVIDIA’s background in graphics and strong presence in deep learning, the institute developed a course on text-to-image diffusion models. However, to avoid training on copyrighted data, the course development had to overcome significant challenges.

During the development process, the team discovered that the CLIP (Contrastive Language-Image Pretraining) algorithm could be used to create text-to-image models without a dedicated text-to-image dataset. CLIP, developed by OpenAI, is a multi-modal model that combines knowledge of English-language concepts with semantic knowledge of images. It leverages a training approach that emphasizes contrasting pairs of images and text to learn a wide range of visual concepts from natural language supervision.

In order to develop this course that reflects the text-to-image capabilities that are prominent in today’s literature on Generative AI but avoids datasets potentially containing copyrighted information, the Deep Learning Institute tested the possibility of using CLIP without a text-to-image dataset. The experiment involved using CLIP’s zero-shot capabilities, which allowed it to perform tasks without task-specific training data. This approach was particularly advantageous because it bypassed the need for a large, labeled dataset, which is often a significant hurdle in AI training due to copyright concerns. By maximizing the cosine similarity between correct image-text pairs and minimizing it for incorrect pairs, CLIP effectively aligned text and image embeddings, enabling the creation of accurate text-to-image models for the students in the course to be able to try hands-on and execute in the notebooks. The experiment showed that CLIP could be used successfully on its own, allowing the creation of a dataset that was both large and robust enough to conduct the training for the course while being copyright-free.

### Participant recruitment

The primary method of recruitment for the AICC Workshop was dissemination through Bridge2AI and CHoRUS sites. The workshop information was posted on the CHoRUS website and the UF AI4Health website, a conference that was held the week following the AICC workshop and was also hosted by the UF College of Medicine. Additional advertisement methods included social media (Instagram and LinkedIn), word of mouth, mass emails sent to the entire Bridge2AI network, advertisement in the Bridge2AI newsletter, and via email dissemination from CHoRUS principal investigators to their sites.

The AICC workshop webpage included a registration link for those interested in attending. Participants of the beginner track paid either a $200 or $150 registration fee for in-person and online participation, respectively. The advanced track registration fee was $300 or $250 for in-person and online participation, respectively.

In alignment with the CHoRUS consortium’s goal of increasing participation from underrepresented groups, 10 travel grants of $1,000 each were awarded to beginner track participants. The web links to apply for these grants were posted on the AICC workshop website and required applicants to submit an updated CV and a personal statement of 500 words maximum describing their professional goals, potential contribution to diversity in medical AI, potential benefit of AICC workshop attendance, financial need, and other funding sources available if they did not receive the grant. The travel grant applications were reviewed and rewarded by the AICC workshop orchestrators.

### Survey design

Following the event, AICC workshop attendees were asked to complete an anonymous post-workshop survey to share workshop feedback and assess their perceived learning gain. This survey was administered via Qualtrics and was designed by a co-lead of the CHoRUS Teaming unit, an assistant professor of health services research, management & policy, and director of evaluation and educational development at the University of Florida. The survey included demographic variables, including gender identity, race and ethnicity, home institution, role and profession, parents’ highest level of education, and whether the respondent grew up in a rural location.

Survey respondents were asked to provide feedback on the clarity of the workshop objectives, satisfaction with workshop networking and mentorship opportunities, whether the networking events resulted in any follow-up plans after the conference, and how future events could be improved [13]. Feedback questions related to objective communication, networking opportunities, and mentoring support were recorded on a scale 1 (Highly Disagree) to 5 (Highly Agree).

Given the tailored nature of the workshop, we developed knowledge and skill assessment questions based on the workshop learning objectives. Specifically, we formulated learning objectives as question prompts and involved program personnel and co-instructors in assessing the content validity of the questions based on the relevance and clarity of the formulations [14]. Respondents were then asked to score their knowledge and skills related to familiarity with Jupyter notebooks and Jupyter Lab environment, ability to develop and execute Python code for manipulating biomedical data, ability to identify Python libraries for biomedical data, and orientation toward multidisciplinary collaboration in medical AI research.

We employed pre-then-post design to address earlier documented concerns about the ability of traditional pre–post designs to capture the shift in participant skill self-assessment. Namely, the participants were asked to assess their skills before the AICC workshop and then asked to reassess their skills at the completion of the workshop. Self-assessment changes depend on a stable “common metric,” but when learners’ understanding shifts, their self-rating criteria may change, leading to response shift bias. This threatens the validity of traditional pre–post designs that attribute changes in self-ratings to educational interventions. The retrospective pre–post method collects data at the same time, comparing retrospective pre- and post-ratings using the same metric. Although objective measures are ideal, the retrospective pre–post method demonstrated validity in identifying learning while being resource-efficient and easy to administer. Skill assessment used the scale of 1 (No Ability) to 5 (Great Ability). The complete questionnaire can be found in Supplementary Materials 3.1.

In addition to the main post-workshop survey, we invited participants to complete a 9-month follow-up survey to assess the workshop’s impact on the participants’ AI/ML research involvement and/or clinical practice. The survey, found in Supplementary Materials 3.2, consisted of 21 multiple-choice and short-response questions on the practical application of skills, networking outcomes, research productivity, and general workshop satisfaction.

### Analysis

Responses from the post-workshop survey were exported from the Qualtrics platform into Microsoft Excel spreadsheets. The data was organized into beginner and advanced track groups. Responses were averaged to create a composite score of self-assessed knowledge before and after the workshop and analyzed using the Wilcoxon signed-rank test. Two-tailed P values of < 0.05 were considered statistically significant.

## Results

### Participant demographics

The 2024 AICC workshop attracted 90 attendees from 14 academic institutions across the United States, with 67 beginner track and 23 advanced track participants. Basic demographic information was collected from the 81 participants who registered in advance and can be found in Supplementary Material 4. The majority of the beginner track group, and nearly two-thirds of the total registrants, were individuals with a clinical background. University faculty, including clinical faculty and PhD/master’s students, were the roles with the highest representation. The CHoRUS travel grant only received ten applications, thus eliminating the need for workshop organizers to select awardees.

The post-workshop survey received a response rate of 44%. Of the 41 workshop attendees who submitted a survey response, 39 participants completed their self-assessment of knowledge before and after the AICC workshop. Twenty-six (63.4%) and twelve (29.2%) of respondents attended the beginner and advanced track, respectively. One respondent, who also did not answer the self-evaluation questions, did not identify their track. Respondent characteristics are presented in Table 1. The top roles included thirteen (31.7%) PhD students, nine (22.0%) university faculty, and seven (17.1%) resident or fellow physicians. Nearly two-thirds (61.0%) of respondents were affiliated with the University of Florida. None of the survey respondents were affiliated with tribal colleges. Twenty-one (51.2%) respondents self-identified as men, thirteen (31.7%) as women, one (2.4%) as transgender, and six (14.6%) did not provide a response. The majority (*n* = 19, 46.3%) of respondents reported Asian/Pacific Islander as their race, followed by twelve (29.3%) White respondents, two (4.9%) Hispanic/Latino, and one (2.4%) multiracial respondent. Seven (17.1%) respondents did not identify their racial or ethnic background.

**Table 1.** Characteristics of AI for Clinical Care post-workshop questionnaire respondents (*N* = 41)

Demographic		Percent	*N*
**Gender Identity**			
	Man	51.2%	21
	Woman	31.7%	13
	Transgender	2.4%	1
	No response	14.6%	6
**Race and Ethnicity**			
	Asian/Pacific Islander	46.3%	19
	Hispanic/Latino	4.9%	2
	Multiracial	2.4%	1
	White	29.3%	12
	No response	17.1%	7
**Role**			
	University Faculty	22.0%	9
	Resident/Fellow Physician	17.1%	7
	Post-Doctoral Researcher	2.4%	1
	PhD Student	31.7%	13
	Medical Student	9.8%	4
	Research Staff	9.8%	4
	Other	7.3%	3
**Parents’ Highest Level of Education**			
	High school or less	9.8%	4
	College degree	17.1%	7
	Advanced or professional degree (PhD, MD, etc.)	56.1%	23
	Other	4.9%	2
	No response	12.2%	5
**Grew up in Rural Area**			
	Yes	12.2%	5
	No	75.6%	31
	No response	12.2%	5
**Institutional Affiliation**			
	University of Florida	61.0%	25
	Emory University	9.8%	4
	Mayo Clinic	4.8%	2
	Other	19.5%	8
	No institutional affiliation	4.9%	2

We followed NIH guidelines to identify seventeen (41.4%) respondents from underrepresented populations in U.S. biomedical and clinical research, which included individuals who reported woman or transgender gender identity, upbringing in a rural area, individuals whose parents’ highest level of education was high school or less, and Black/African American, Native American, or Hispanic or Latino race or ethnicity [15].

### Post-workshop survey results

Mean scores from twenty-six beginner track respondents identified “Familiarity with and application of large language models (LLMs)” as the learning objective with the lowest baseline knowledge (mean of 1.85) and “Discuss the importance of multidisciplinary collaboration for the advancement of AI” as the highest baseline score (mean of 2.50). The self-reported knowledge gain of all six beginner track learning objectives is presented in Table 2. The largest increase was found in the identification of important Python libraries for biomedical data science (difference in mean of 1.38), and the highest average post-workshop score was in the importance of multidisciplinary collaboration for the advancement of AI (mean of 3.65). In total, the average of all learning objectives increased from “Some ability” to “Moderate to Good ability.” Wilcoxon signed-rank test indicated that post-workshop knowledge scores were statistically significantly higher than pre-workshop scores, *Z* = −4.17, *p* < 0.01, with a median score of 3.43 for the post-workshop compared to 2.16 for the pre-workshop scores.

**Table 2.** Scores for before and after workshop for each question; beginner track

Learning Objective	Mean Score Before	Mean Score After	Difference in Mean
Get familiar and apply LLMs with Jupyter notebooks	1.85	3.15	1.31
Navigate the Jupyter lab environment and create Jupyter notebooks	2.15	3.31	1.15
Explain the rules that govern Python variables, loops, conditionals, and functions	2.31	3.62	1.31
Develop and execute Python code for manipulating biomedical data	2.31	3.50	1.16
Identify the important Python libraries for biomedical data science	2.08	3.46	1.38
Discuss the importance of multidisciplinary collaboration for advancing medical AI	2.50	3.65	1.15

Mean scores from twelve advanced track respondents, presented in Table 3, showed an understanding of controlling image output with context embeddings and generation of images from English text prompts using CLIP to be the objectives with the highest baseline understanding (mean of 3.00 for both). Improving the quality of generated images with denoising diffusion and comparing denoising diffusion probabilistic models (DDPMs) with denoising diffusion implicit models (DDIMs) were the objectives with the lowest baseline understanding (mean of 2.83 for both). The highest increase in self-reported knowledge was found in building a U-Net to generate images from pure noise (difference in mean of 1.25). In total, the average of all learning objectives increased from “moderate ability” to “good ability.” Wilcoxon signed-rank test indicated that post-workshop knowledge scores were statistically significantly higher than pre-workshop scores, *Z* = −2.08, *p* < 0.05, with a median score of 4.07 for the post-workshop compared to 2.92 for the pre-workshop scores.

**Table 3.** Scores for before and after workshop per learning objective; advanced track

Learning Objective	Mean Score Before	Mean Score After	Difference in Mean
Build a *U*-Net to generate images from pure noise	2.92	4.17	1.25
Improve the quality of generated images with the Denoising Diffusion process	2.83	4.00	1.17
Compare Denoising Diffusion Probabilistic Models (DDPMs) with Denoising Diffusion Implicit Models (DDIMs)	2.83	4.00	1.67
Control the image output with context embeddings	3.00	4.08	1.08
Generate images from English text-prompts using Contrastive Language-Image Pretraining (CLIP).	3.00	4.08	1.08

### Workshop feedback

On a scale of 1 to 5, respondents rated their satisfaction with communication of workshop objectives as an average of 4.39 (SD: 1.09), networking opportunities with CHoRUS investigators and trainees as 4.12 (SD: 1.14), and the opportunity to receive mentoring and support as 4.02 (SD: 1.17). The mean of all three metrics was 4.18, which corresponds to participants being “satisfied” with these aspects of the AICC workshop. All forty-one post-workshop survey respondents provided feedback on these three metrics.

Written response feedback from advanced track participants was largely positive. Suggestions to improve future advanced track workshops were the inclusion of additional concrete examples of future applications and added “FIXME” comments to encourage further interaction with the code. Seven out of the twelve advanced track respondents reported that the workshop resulted in plans to follow-up after the conference (e.g., continual mentoring, research collaboration, talk, visit), whereas only three of twenty-six beginner track respondents reported this. Respondents of the beginner track provided greater workshop feedback. First, participants identified a need for an intermediate course. While some respondents reported that the beginner course was too basic and repeated elements from the pre-workshop self-paced courses, others found the workshop difficult to follow without a background in coding. Two respondents identified technical issues completing the self-paced courses as Windows users.

Several respondents from both tracks commented on the lack of structure in the networking events. Suggestions to improve this aspect included assigning mentors to a group of mentees, dividing the lunch room into groups based on research interests, and having workshop organizers lead a rotation of mentors and mentees to give the mentees the opportunity to talk to additional people. Furthermore, the lack of mentoring or networking opportunities for online participants was identified as a shortcoming of the hybrid workshop format.

### Follow-up survey results

To evaluate the long-term impact of the AICC workshop, a follow-up survey was distributed approximately 9 months after the event. Despite the low response rate (*N* = 6), the survey provided preliminary insight into how a sample of participants have applied the workshop content. Respondents included two clinical faculty, two PhD students, and one tenure- or research-track faculty member. One respondent selected “other” but did not clarify their position in the provided text box. A table containing the multiple-choice data is presented in Supplementary Materials 3.3.

Five out of six respondents (∼83%) reported being at least “somewhat confident” in their ability to implement AI tools or techniques in their work. All six of the follow-up survey respondents reported applying the AI tools and techniques to their work to some extent, with half rating their application as “somewhat” and half as “moderately.” Of those who reported AI application in their clinical practice, three reported this as “moderate” and one as “significant.” The examples of this application included the use of ML to predict postoperative complications in high-risk patients, use of the workshop code examples in a research project, and the development of AI models and LLMs with retrospective datasets.

No respondents reported any formal research collaboration resulting directly from the AICC workshop, but half of the respondents (*N* = 3) indicated that they had continued connections with individuals they met at the event. While the extent of the workshop’s impact varied – two responding “no impact,” two “slight impact,” and two “moderate impact” – the majority (5 of 6) respondents have either published or have plans to publish papers related to AI in clinical care.

All six of the respondents rated the workshop’s value as at least “somewhat valuable” and all but one said they would recommend it to their colleagues in the healthcare field. Barriers to AI implementation included time constraints, limited institutional support, and access to appropriate datasets or HIPAA-compliant servers. Participants suggested that explicit guidance on data access, higher integration of workshop tracks, and additional workshops or hackathon events throughout the year could help support sustained collaboration and application of AICC content. Finally, one participant commented on the limited networking opportunities for the online participants.

## Discussion

The AICC workshop was meticulously designed to be relevant to clinicians and clinical researchers to address the critical need for an AI-competent healthcare workforce. It employed a mix of online pre-workshop training from the UF Office of Professional and Workforce Development with hands-on experiential learning delivered by leading experts in medical AI. This workshop established a core AI vocabulary to prepare clinicians for future interactions with AI and bypassed the complex mathematical foundations of machine learning techniques. Instead, participants were presented with clinical applications of AI to address real-world problems, exploring its context of use and the indications of when, how, and why to use AI. This approach treated AI much like a clinical tool or medical device, preparing clinicians for the imminent availability of AI clinical decision support systems in their clinical practice.

A fundamental barrier to AI adoption remains the lack of basic AI knowledge among healthcare professionals [9]. Without adequate understanding, professionals cannot effectively assess the level of training required for competency or evaluate the appropriateness of AI applications in their practice. Many healthcare professionals report they lack even a basic understanding of AI principles, creating uncertainty about its applications and limitations [5]. By furthering clinicians’ ability to recognize AI/ML models and question their development and application, the workshop’s curriculum builds the foundational knowledge to support informed, critical engagement with algorithmic outputs – an important factor in mitigating potential biases in the application of medical AI. Moreover, the workshop’s mix of AI novices and experts promoted networking, collaboration, and mentorship and gave engineers and data scientists the opportunity to connect with the end-users of their medical AI tools and gain insight into their unique needs.

Following participation in the AICC workshop, post-workshop survey respondents expressed satisfaction with the workshop experience and reported significant improvements in their understanding of AI and machine learning systems, as well as increased confidence in applying these technologies. We observed notable gains in self-reported knowledge and skills across all eleven objectives in both the beginner and advanced tracks. For the beginner track, the highest learning gain was reported in identifying Python libraries for biomedical data science. In the advanced track, the most significant learning gains were seen in improving the quality of generated images with denoising diffusion and comparing DDPMs with DDIMs. Approximately one quarter of the post-workshop survey respondents shared that the workshop networking events resulted in follow-up plans after the conference, including continued mentorship, research collaborations, meetings, and presentations.

In the follow-up survey distributed approximately nine months after the event, all respondents (*N* = 6) reported application of workshop tools and techniques into their work, and a majority (5 of 6) have initiated or participated in new AICC collaborations or projects since the workshop. However, while most (5 of 6) have authored or intend to author publications or presentations on AICC topics, no respondents reported any new research collaborations resulting from the workshop.

The present study details the design and analysis of a one-day hybrid workshop and contributes to the systematic knowledge on developing and evaluating workforce skill development programs. While many of these events go unreported, sharing their outcomes is crucial for disseminating best practices and guiding the creation of successful educational initiatives that effectively measure impact, knowledge transfer, and delivery of intended benefits [16]. In this instance, the AICC workshop assessed knowledge based on the learning objectives of its two tracks, asking participants to evaluate their skills before and after the event and share its long-term impacts in a follow-up survey. Given the already overburdened medical curriculum and the challenges of incorporating digital health education into healthcare [7], the AICC workshop was strategically designed to confer high-yield skill development as a single-day event held on a Sunday.

Despite these promising knowledge gains, this study is limited to a sample size of 39 (43%) respondents who completed their before-and-after knowledge assessments out of 90 AICC workshop attendees. While 52% of advanced track participants completed the post-workshop survey, the beginner track only received a response rate of 39%. This can be attributed to the beginner track’s dense workshop agenda that consisted of 6 Jupyter Notebooks, 3 presentations, and 1 LLM Copilot Demo. Participants of the advanced track were given the opportunity to complete the survey during the event, unlike beginner track participants. Moreover, only approximately 7% of workshop attendees completed the follow-up survey, most likely due to the time elapsed since the workshop, lack of incentive for completion, and competing clinical and academic demands. Furthermore, only six participants completed a nine-month follow-up. Our findings may not describe the experiences of all AICC workshop attendees. Methodologically, future workshops may benefit from administering evaluation surveys before participants leave the workshop and using persistent identifiers, like ORCID id, to collect follow up data without reliance on participant surveys.

Furthermore, this study used a pre-then-post design where participants were asked to assess their knowledge and skills before and after the AICC workshop in a single, combined post-workshop evaluation questionnaire. While this methodological design allowed us to collect anonymous results and forgo the need to match pre- and post-workshop surveys, the results are subject to the effects of recall and social desirability biases as a result. To lessen social desirability bias, future workshops may benefit by collecting demographic information with a separate survey to reduce the likelihood of identifying participants’ feedback by their less common institutions, gender, racial or ethnic identity, or role. This survey could be implemented during workshop registration or as an elective pre-workshop survey. However, this would limit the ability to explore and compare reported outcomes between demographic groups. In addition, the current workshop evaluation could be improved with the addition of questions about the perceived relevance of the content, how they plan to apply what they learned in the future, and objective skill assessments beyond self-reporting [16]. Several beginner track participants identified the absence of an intermediate track as a limitation, noting the repetition of content from the required pre-workshop online course in the workshop. Additionally, beginner track attendees shared that the afternoon session, which covered three Jupyter Notebooks and three thirty-minute presentations, felt rushed. However, participants expressed high satisfaction with the online pre-workshop course, which suggests that future AICC workshops could be optimized by condensing or omitting some Jupyter Notebooks to allow more time for hands-on activities. Moreover, the advanced track’s specific topic on Generative AI with diffusion models and requirement of experience with PyTorch led to the exclusion of some otherwise suitable candidates, including an associate professor who conducts medical AI research. The inclusion of pre-workshop coursework in the advanced track should thus be considered for future AICC workshops to decrease the disparity between beginner and advanced groups.

Participants who provided feedback on the networking events shared criticisms that they lacked structure and depended on mentees to initiate contact with mentors or connect with others based on who they happened to sit with at lunch. A previous study found that effective leadership enhances individual development and self-reported learning in similar events [4]. Therefore, to improve future AICC workshops, networking events could assign mentors to a small group of mentees based on research interests collected prior to the workshop. Future networking events should also consider the involvement of online participants. Additionally, participants suggested practical improvements such as providing sticky notes for exchanging contact information, organizing rotation sessions led by the event organizers to ensure mentee engagement with multiple mentors, and setting up designated tables for different research interests during lunch. These changes could foster more meaningful interactions and better align networking opportunities with participants’ professional interests. To reinforce learning and acquired skills, future efforts could also include other AICC-themed events such as hackathons or datathons held throughout the year. Prior studies have shown that such events enhance affective learning and promote interdisciplinary collaboration [4,7]. Thus, such events could provide participants with hands-on practice, extend the relationships formed during previous events, and foster a sense of community that supports continued engagement. The addition of such events may also address the lack of engagement we received from the follow-up survey, especially if dedicated time is provided in these later events for participants to complete it.

## Conclusion

The AICC workshop offered beginner and advanced track skills and professional development in AI for a diverse group of trainees, physicians, and early-stage investigators in medicine. Beginner track attendees participated in the AI Bootcamp, and advanced track attendees partook in the Generative AI with Diffusion Models Workshop led by NVIDIA instructors. Attendees received the opportunity to receive mentorship and advice from mentors from prestigious research institutions, which resulted in plans for future plans for AI-related activities, research collaborations, meetings, and meaningful connections. Post-workshop survey respondents showed significant improvements in their knowledge of workshop learning objectives. Future events should consider the inclusion of an intermediate track or a pre-workshop course for the advanced track, increased advertisement to tribal colleges and underrepresented groups, and structured networking sessions or mentoring groups.

## Supporting information

10.1017/cts.2025.10156.sm001 Davidson et al. supplementary material 1 Davidson et al. supplementary material

10.1017/cts.2025.10156.sm002 Davidson et al. supplementary material 2 Davidson et al. supplementary material

10.1017/cts.2025.10156.sm003 Davidson et al. supplementary material 3 Davidson et al. supplementary material

10.1017/cts.2025.10156.sm004 Davidson et al. supplementary material 4 Davidson et al. supplementary material

10.1017/cts.2025.10156.sm005 Davidson et al. supplementary material 5 Davidson et al. supplementary material

## Supplementary material

The supplementary material for this article can be found at https://doi.org/10.1017/cts.2025.10156
