# Supplementary material for: Building AI competence in the healthcare workforce with the AI for clinical care workshop: A Bridge2AI for clinical CHoRUS project
Source: J Clin Transl Sci. 2025 Oct 3;9(1):e243. doi: 10.1017/cts.2025.10156 (PMC12695489; doi:10.1017/cts.2025.10156)
Supplement: Davidson et al. supplementary material 1 — Davidson et al. supplementary material [file S2059866125101568sup001.docx]

**Building AI Competence in the Healthcare Workforce with the AI for Clinical Care Workshop: a Bridge2AI for Clinical CHoRUS Project**

Andrea E. Davidson, BS, Aiden Jose, Benjamin Shickel, PhD, Kaleb E. Smith, PhD, Parisa Rashidi, PhD, Yulia Levites Strekalova, PhD, MBA, Azra Bihorac, MD, MS

Supplemental Material 1: AICC Workshop Agenda

| 7:30 AM | Check-In, Breakfast, and Brief Fun Networking Activity | |
| --- | --- | --- |
| 8:30 AM | Morning Concurrent Workshop Tracks | |
| 8:30 AM – 12:30 PM | Beginner Track:   1. Welcome & Setup 2. Jupyter Notebook #1: Introduction to Python (Group) 3. Jupyter Notebook #2: Biomedical Data Analysis (Group) 4. LLM Copilot Demo 5. Jupyter Notebook #3: Introduction to Version Control (Individual) | Advanced Track:   1. Introduction 2. From U-Nets to Diffusion 3. Control with Context 4. Text-to-Image with CLIP |
| 12:30 PM | Networking Lunch | |
| 1:30 PM | Afternoon Concurrent Workshop Tracks | |
| 1:30 PM – 5:30 PM | Beginner Track:   1. Jupyter Notebook #4: Machine Learning for Clinical Care (Group) 2. Jupyter Notebook #5: Introduction to Deep Learning (Group) 3. Jupyter Notebook #6: Clinical AI Ethics (Individual) 4. Presentation by Andrew Williams, PhD, Tufts University: Introduction to Data Standards 5. Presentation by Rishikesan Kamaleswaran, PhD, Emory University: Introduction to Physiological Waveforms 6. Presentation by Tyler Loftus, MD, PhD, University of Florida: Introduction to Clinical Applications of AI | Advanced Track:   1. State-of-the-art Models 2. Final Review |
