# Supplementary material for: Building AI competence in the healthcare workforce with the AI for clinical care workshop: A Bridge2AI for clinical CHoRUS project
Source: J Clin Transl Sci. 2025 Oct 3;9(1):e243. doi: 10.1017/cts.2025.10156 (PMC12695489; doi:10.1017/cts.2025.10156)
Supplement: Davidson et al. supplementary material 2 — Davidson et al. supplementary material [file S2059866125101568sup002.docx]

**Building AI Competence in the Healthcare Workforce with the AI for Clinical Care Workshop: a Bridge2AI for Clinical CHoRUS Project**

Andrea E. Davidson, BS, Aiden Jose, Benjamin Shickel, PhD, Kaleb E. Smith, PhD, Parisa Rashidi, PhD, Yulia Levites Strekalova, PhD, MBA, Azra Bihorac, MD, MS

Supplementary Materials 2: Advanced Track Eligibility Questionnaire

1. What is your current computer hardware?
   1. Do you use a multi-GPU cluster/workstation?
2. What is your current AI pipeline for medical images?
3. Do you feel bottlenecked from compute power?
   1. Would multi-GPU work flow allow you to do bigger/better research in your current research focus?
4. What DL framework do you use for your research?
5. Do you have a good understanding of PyTorch?
6. What value do you see in having state-of-the-art AI models ready to be used on your data with little to no coding changes?
7. How do you annotate your medical images?
8. Do you have deployment applications in clinical/medical setting with your AI models?
9. Do you often find models on open source repos and utilize their pretrain weights instead of training from scratch?
   1. Do you make your models completely custom and not use validated and published AI models?
