# Supplementary material for: Building AI competence in the healthcare workforce with the AI for clinical care workshop: A Bridge2AI for clinical CHoRUS project
Source: J Clin Transl Sci. 2025 Oct 3;9(1):e243. doi: 10.1017/cts.2025.10156 (PMC12695489; doi:10.1017/cts.2025.10156)
Supplement: Davidson et al. supplementary material 3 — Davidson et al. supplementary material [file S2059866125101568sup003.docx]

**Building AI Competence in the Healthcare Workforce** **with the AI for Clinical Care Workshop: a Bridge2AI for Clinical CHoRUS Project**

Andrea E. Davidson, BS, Aiden Jose, Benjamin Shickel, PhD, Kaleb E. Smith, PhD, Parisa Rashidi, PhD, Yulia Levites Strekalova, PhD, MBA, Azra Bihorac, MD, MS

Supplementary Material 3.1: Post-Workshop Questionnaire

Participant Demographics

1. What best describes your role?
   1. University faculty
   2. Resident/Fellow
   3. Post-Doc
   4. Medical student
   5. PhD student
   6. Research staff
   7. CHoRUS MPI
   8. Other: ____
2. What is your home institution? [short response]
3. What is your gender identity?
   1. Man
   2. Woman
   3. Transgender
   4. Other: _____
4. What best describes your race and ethnicity?
   1. African American/Black
   2. Asian or Pacific Islander
   3. Caribbean descent
   4. Hispanic or Latino
   5. Multiracial
   6. Native American or American Indian
   7. White
   8. Other: ____
5. What is the highest level of education of your parents?
   1. High school or less
   2. College degree
   3. Advanced or professional degree (PhD, MD, etc)
   4. Other: _____
6. Did you grow up in a rural area?
   1. Yes
   2. No

Workshop Feedback

1. Objectives were clearly communicated throughout the event sessions and activities.
   1. Highly Dissatisfied
   2. Dissatisfied
   3. Neutral
   4. Satisfied
   5. Highly Satisfied
2. The AI for Clinical Care Workshop provided an opportunity for me to network with CHoRUS Investigators and trainees.
   1. Highly Dissatisfied
   2. Dissatisfied
   3. Neutral
   4. Satisfied
   5. Highly Satisfied
3. The workshop provided an opportunity for me to receive mentoring advice and support.
   1. Highly Dissatisfied
   2. Dissatisfied
   3. Neutral
   4. Satisfied
   5. Highly Satisfied
4. Which AI for Clinical Care Workshop track did you attend?
   1. Beginner track
   2. Advanced track
5. How can we improve this workshop in the future? [open response]
6. Have you attended the mentoring lunch session?
   1. Yes
   2. No
7. How can we enhance the opportunities for mentor-trainee engagement in the future? [open response]
8. Please describe below if networking during the AI for Clinical Care Workshop resulted in any plans to follow-up after the conference (e.g., continual mentoring, research collaboration, talk visit, etc.) [open response]
9. We are planning an AI for Clinical Care Data Challenge. What educational and mentoring activities should we consider for the future data challenge? [open response]

Beginner Track Questions

1. How would you assess your knowledge and skills listed below BEFORE the AI for Clinical Care Workshop? - Get familiar and apply LLMs with Jupyter notebooks
   1. No ability
   2. Some ability
   3. Moderate ability
   4. Good ability
   5. Great ability
2. How would you assess your knowledge and skills listed below BEFORE the AI for Clinical Care Workshop? - Navigate the Jupyter Lab environment and create Jupyter notebooks
   1. No ability
   2. Some ability
   3. Moderate ability
   4. Good ability
   5. Great ability
3. How would you assess your knowledge and skills listed below BEFORE the AI for Clinical Care Workshop? - Explain the rules that govern Python variables, loops, conditionals, and functions
   1. No ability
   2. Some ability
   3. Moderate ability
   4. Good ability
   5. Great ability
4. How would you assess your knowledge and skills listed below BEFORE the AI for Clinical Care Workshop? - Develop and execute Python code for manipulating biomedical data
   1. No ability
   2. Some ability
   3. Moderate ability
   4. Good ability
   5. Great ability
5. How would you assess your knowledge and skills listed below BEFORE the AI for Clinical Care Workshop? - Identify the important Python libraries for biomedical data science
   1. No ability
   2. Some ability
   3. Moderate ability
   4. Good ability
   5. Great ability
6. How would you assess your knowledge and skills listed below BEFORE the AI for Clinical Care Workshop? - Discuss the importance of multidisciplinary collaboration for advancing medical AI
   1. No ability
   2. Some ability
   3. Moderate ability
   4. Good ability
   5. Great ability
7. How would you assess your knowledge and skills listed below AFTER the AI for Clinical Care Workshop? - Get familiar and apply LLMs with Jupyter notebooks
   1. No ability
   2. Some ability
   3. Moderate ability
   4. Good ability
   5. Great ability
8. How would you assess your knowledge and skills listed below AFTER the AI for Clinical Care Workshop? - Navigate the Jupyter Lab environment and create Jupyter notebooks
   1. No ability
   2. Some ability
   3. Moderate ability
   4. Good ability
   5. Great ability
9. How would you assess your knowledge and skills listed below AFTER the AI for Clinical Care Workshop? - Explain the rules that govern Python variables, loops, conditionals, and functions
   1. No ability
   2. Some ability
   3. Moderate ability
   4. Good ability
   5. Great ability
10. How would you assess your knowledge and skills listed below AFTER the AI for Clinical Care Workshop? - Develop and execute Python code for manipulating biomedical data
    1. No ability
    2. Some ability
    3. Moderate ability
    4. Good ability
    5. Great ability
11. How would you assess your knowledge and skills listed below AFTER the AI for Clinical Care Workshop? - Identify the important Python libraries for biomedical data science
    1. No ability
    2. Some ability
    3. Moderate ability
    4. Good ability
    5. Great ability
12. How would you assess your knowledge and skills listed below AFTER the AI for Clinical Care Workshop? - Discuss the importance of multidisciplinary collaboration for advancing medical AI
    1. No ability
    2. Some ability
    3. Moderate ability
    4. Good ability
    5. Great ability

Advanced Track Questions

1. How would you assess your knowledge and skills listed below BEFORE the AI for Clinical Care Workshop? - Build a U-Net to generate images from pure noise
   1. No ability
   2. Some ability
   3. Moderate ability
   4. Good ability
   5. Great ability
2. How would you assess your knowledge and skills listed below BEFORE the AI for Clinical Care Workshop? - Improve the quality of generated images with the Denoising Diffusion process
   1. No ability
   2. Some ability
   3. Moderate ability
   4. Good ability
   5. Great ability
3. How would you assess your knowledge and skills listed below BEFORE the AI for Clinical Care Workshop? - Compare Denoising Diffusion Probabilistic Models (DDPMs) with Denoising Diffusion Implicit Models (DDIMs)
   1. No ability
   2. Some ability
   3. Moderate ability
   4. Good ability
   5. Great ability
4. How would you assess your knowledge and skills listed below BEFORE the AI for Clinical Care Workshop? - Control the image output with context embeddings
   1. No ability
   2. Some ability
   3. Moderate ability
   4. Good ability
   5. Great ability
5. How would you assess your knowledge and skills listed below BEFORE the AI for Clinical Care Workshop? - Generate images from English text-prompts using CLIP
   1. No ability
   2. Some ability
   3. Moderate ability
   4. Good ability
   5. Great ability
6. How would you assess your knowledge and skills listed below AFTER the AI for Clinical Care Workshop? - Build a U-Net to generate images from pure noise
   1. No ability
   2. Some ability
   3. Moderate ability
   4. Good ability
   5. Great ability
7. How would you assess your knowledge and skills listed below AFTER the AI for Clinical Care Workshop? - Improve the quality of generated images with the Denoising Diffusion process
   1. No ability
   2. Some ability
   3. Moderate ability
   4. Good ability
   5. Great ability
8. How would you assess your knowledge and skills listed below AFTER the AI for Clinical Care Workshop? - Compare Denoising Diffusion Probabilistic Models (DDPMs) with Denoising Diffusion Implicit Models (DDIMs)
   1. No ability
   2. Some ability
   3. Moderate ability
   4. Good ability
   5. Great ability
9. How would you assess your knowledge and skills listed below AFTER the AI for Clinical Care Workshop? - Control the image output with context embeddings
   1. No ability
   2. Some ability
   3. Moderate ability
   4. Good ability
   5. Great ability
10. How would you assess your knowledge and skills listed below AFTER the AI for Clinical Care Workshop? - Generate images from English text-prompts using CLIP
    1. No ability
    2. Some ability
    3. Moderate ability
    4. Good ability
    5. Great ability

Supplementary Materials 3.2: Follow-Up Survey

*Section 1: Practical Application of Skills*

1. Which workshop tract did you attend?
   1. Beginner track (AI for Clinical Care Workshop Bootcamp)
   2. Advanced track (NVIDIA Generative AI with Diffusion Models Course)
2. Which of the following best describes your current position?
   1. Undergraduate student
   2. Master's student
   3. Medical student
   4. Pharmacy Student
   5. PhD student
   6. Post doctoral researcher (e.g., research fellow, post-doc)
   7. Research staff (e.g., data scientist, laboratory technician, research assistant)
   8. Resident or fellow physician
   9. Tenure- or research-track faculty
   10. Clinical faculty (attending physician)
   11. Other: ___________________
3. To what extent have you applied the AI tools and techniques learned in the workshop to your work?
   1. Not at all
   2. Somewhat
   3. Moderately
   4. Significantly
   5. Fully integrated in my practice
4. If you are a clinician, to what extent have you applied the AI tools and techniques learned in the workshop to your clinical practice?
   1. Not at all
   2. Somewhat
   3. Moderately
   4. Significantly
   5. Fully integrated in my practice
   6. Not applicable
5. Can you provide specific examples of how you’ve used AI tools or techniques in your work or clinical practice since the workshop?
   1. [Short Response]
6. How confident are you in your ability to implement AI solutions in clinical care following the workshop?
   1. Not confident
   2. Somewhat confident
   3. Confident
   4. Very confident
7. What challenges or barriers have you encountered when attempting to implement AI tools or techniques in your work? *Please describe any specific obstacles (e.g., technical, organizational, time-related) you have faced.*
   1. [Short Response]

*Section 2: Collaboration and Networking Opportunities*

1. Did the AICC Workshop result in any research collaborations?
   1. Yes
   2. No
2. Please describe the project and its status.
   1. [Short Response]
3. Have you initiated or participated in any new collaborations or projects since the workshop related to AI in clinical care?
   1. Yes, new collaborations initiated
   2. Yes, participated in existing collaborations
   3. No new collaborations, but have maintained existing ones
   4. No collaborations or projects related to AI
4. If you initiated or joined new collaborations, please describe them. Include the scope and current status of any AI-related projects or partnerships.
   1. [Short Response]
5. Have you continued any relationships or networks established during the workshop (e.g., with participants, mentors, or industry professionals)?
   1. Yes
   2. No
   3. Partially (e.g., occasional contact, but no sustained collaboration)

*Section 3: Research Productivity and Publications*

1. Since attending the workshop, have you authored or co-authored any publications, presentations, or papers related to AI in clinical care?
   1. Yes
   2. No
   3. Planning to, but not published yet
2. [If yes] Please provide the titles and publication details (e.g., journal name, conference, date) of any research outputs related to AI in clinical care. If you prefer to remain anonymous, please enter the number of publications, presentations, or papers you have authored in the field since attending the workshop.
   1. [Short Response]
3. How has attending the workshop impacted your research productivity in the field of AI in clinical care?
   1. No impact
   2. Slight impact
   3. Moderate impact
   4. Significant impact
4. Do you foresee any future research or AI-driven clinical care projects stemming from your participation in the workshop?
   1. Yes
   2. No
   3. Unsure
5. If yes, what are the main areas or topics you plan to focus on in future research, or AI-related clinical care initiatives?
   1. [Short Response]

*Section 4: Overall Impact and Satisfaction*

1. Overall, how valuable was the workshop in equipping you with skills and knowledge to advance AI in clinical care?
   1. Not valuable
   2. Somewhat valuable
   3. Valuable
   4. Extremely valuable
2. Would you recommend this workshop to colleagues or peers in the healthcare field?
   1. Yes
   2. No
3. What additional support, resources, or follow-up would have helped you further implement AI tools or continue the collaborations started at the workshop?
   1. [Short Response]
4. Do you have any additional feedback you would like to share with the workshop organizers?
   1. [Short Response]

3.3 Follow-Up Survey Results Table

| **Survey Question** | **Multiple Choice Options** | | **N** | **Approximate**  **Percent** |
| --- | --- | --- | --- | --- |
| Which of the following best describes your current position? | |  |  |  |
|  |  | Clinical faculty (attending physician) | 2 | 33% |
|  |  | PhD Student | 2 | 33% |
|  |  | Tenure- or research-track faculty | 1 | 17% |
|  |  | Other | 1 | 17% |
| Which workshop tract did you attend? | |  |  |  |
|  |  | Beginner track (AI for Clinical Care Workshop Bootcamp) | 1 | 17% |
|  |  | Advanced track (NVIDIA Generative AI with Diffusion Models Course) | 5 | 83% |
| **Practical Application of Skills** | | | | |
| To what extent have you applied the AI tools and techniques learned in the workshop to your work? | |  |  |  |
|  |  | Not at all | 0 | 0% |
|  |  | Somewhat | 3 | 50% |
|  |  | Moderately | 3 | 50% |
|  |  | Significantly | 0 | 0% |
|  |  | Fully integrated into my practice | 0 | 0% |
| If you are a clinician, to what extent have you applied the AI tools and techniques learned in the workshop to your clinical practice? | |  |  |  |
|  |  | Not at all | 1 | 17% |
|  |  | Somewhat | 0 | 0% |
|  |  | Moderately | 3 | 50% |
|  |  | Significantly | 1 |  |
|  |  | Fully integrated into my practice | 0 | 0% |
|  |  | Not applicable | 1 | 17% |
| How confident are you in your ability to implement AI solutions in clinical care following the workshop? | |  |  |  |
|  |  | Not confident | 1 | 17% |
|  |  | Somewhat confident | 3 | 50% |
|  |  | Confident | 2 | 33% |
|  |  | Very confident | 0 | 0% |
| **Collaboration and Networking Opportunities** | | | | |
| Did the AICC Workshop result in any research collaborations? | | Yes | 0 | 0% |
|  |  | No | 6 | 100% |
| Have you initiated or participated in any new collaborations or projects since the workshop related to AI in clinical care? | |  |  |  |
|  |  | Yes, new collaborations initiated | 1 | 17% |
|  |  | Yes, participated in existing collaborations | 2 | 33% |
|  |  | No new collaborations, but have maintained existing ones | 2 | 33% |
|  |  | No collaborations or projects related to AI | 1 | 17% |
| Have you continued any relationships or networks established during the workshop (e.g., with participants, mentors, or industry professionals)? | |  |  |  |
|  |  | Yes | 1 | 17% |
|  |  | No | 3 | 50% |
|  |  | Partially (e.g., occasional contact, but no sustained collaboration) | 2 | 33% |
| **Research Productivity and Publications** | | | | |
| Since attending the workshop, have you authored or co-authored any publications, presentations, or papers related to AI in clinical care? | |  |  |  |
|  |  | Yes | 2 | 33% |
|  |  | No | 1 | 17% |
|  |  | Planning to, but not published yet | 3 | 50% |
| How has attending the workshop impacted your research productivity in the field of AI in clinical care? | |  |  |  |
|  |  | No impact | 2 | 33% |
|  |  | Slight impact | 2 | 33% |
|  |  | Moderate impact | 2 | 33% |
|  |  | Significant impact | 0 | 0% |
| Do you foresee any future research or AI-driven clinical care projects stemming from your participation in the workshop? | |  |  |  |
|  |  | Yes | 3 | 50% |
|  |  | No | 0 | 0% |
|  |  | Unsure | 3 | 50% |
| **Overall Impact and Satisfaction** | | | | |
| Overall, how valuable was the workshop in equipping you with skills and knowledge to advance AI in clinical care? | |  |  |  |
|  |  | Not valuable | 0 | 0% |
|  |  | Somewhat valuable | 2 | 33% |
|  |  | Valuable | 2 | 33% |
|  |  | Extremely valuable | 1 | 17% |
| Would you recommend this workshop to colleagues or peers in the healthcare field? | |  |  |  |
|  |  | Yes | 5 | 83% |
|  |  | No | 1 | 17% |
