# Supplementary material for: Building AI competence in the healthcare workforce with the AI for clinical care workshop: A Bridge2AI for clinical CHoRUS project
Source: J Clin Transl Sci. 2025 Oct 3;9(1):e243. doi: 10.1017/cts.2025.10156 (PMC12695489; doi:10.1017/cts.2025.10156)
Supplement: Davidson et al. supplementary material 4 — Davidson et al. supplementary material [file S2059866125101568sup004.docx]

**Building AI Competence in the Healthcare Workforce with the AI for Clinical Care Workshop: a Bridge2AI for Clinical CHoRUS Project**

Andrea E. Davidson, BS, Aiden Jose, Benjamin Shickel, PhD, Kaleb E. Smith, PhD, Parisa Rashidi, PhD, Yulia Levites Strekalova, PhD, MBA, Azra Bihorac, MD, MS

Supplementary Material 4.1: Total Workshop Registrant Demographics

| **Demographic** | | **N** | **Percent** |
| --- | --- | --- | --- |
| **Sex** | Male | 49 | 60.4% |
|  | Female | 32 | 39.5% |
| **Role** |  |  |  |
|  | University Faculty  (including clinical faculty) | 23 | 28.4% |
|  | Resident/Fellow Physician | 7 | 8.6% |
|  | Non-Academic Clinician:  Physician (n=6), PA (n=1) | 7 | 8.6% |
|  | Post-Doctoral Researcher or Research Fellow | 5 | 6.2% |
|  | PhD and Masters Students | 12 | 14.8% |
|  | Medical college student:  Medical students (n=6), Nursing student (n=1), PharmD student (n=1) | 8 | 9.9% |
|  | Research Staff | 12 | 14.8% |
|  | Pre-Medical Students | 3 | 3.7% |
|  | Other | 4 | 4.9% |
| **Institutions Represented** |  |  |  |
|  | University of Florida | 49 | 60.5% |
|  | Emory University | 5 | 6.2% |
|  | Mayo Clinic | 5 | 6.2% |
|  | Marshall University | 1 | 1.2% |
|  | Massachusetts General Brigham | 1 | 1.2% |
|  | University of Colorado | 2 | 2.5% |
|  | Benjamin Franklin Cummings Institute of Technology | 1 | 1.2% |
|  | University of Alabama Birmingham | 1 | 1.2% |
|  | University of Michigan | 1 | 1.2% |
|  | University of South Florida | 1 | 1.2% |
|  | University of California Los Angeles | 2 | 2.5% |
|  | Brown University | 1 | 1.2% |
|  | Rutgers University | 1 | 1.2% |
|  | Yale | 1 | 1.2% |
|  | Not affiliated with a US academic institution | 9 | 11.1% |
| **Background** |  |  |  |
|  | Clinical | 50 | 61.7% |
|  | Data Science or Engineering | 31 | 38.3% |

| **Demographic** | | **Beginner Track**  **(N)** | **Advanced Track (N)** |
| --- | --- | --- | --- |
| **Sex** | Male | 36 | 13 |
|  | Female | 27 | 5 |
| **Role** |  |  |  |
|  | University Faculty  (includes clinical faculty) | 19 | 4 |
|  | Resident/Fellow Physician | 6 | 1 |
|  | Non-Academic Clinician:  Physician (n=6), PA (n=1) | 7 | 0 |
|  | Post-Doctoral Researcher or Research Fellow | 4 | 1 |
|  | PhD and Masters Students | 6 | 6 |
|  | Medical college student:  Medical students (n=6), Nursing student (n=1), PharmD student (n=1) | 7 | 1 |
|  | Research Staff | 7 | 5 |
|  | Pre-Medical Students | 3 | 0 |
|  | Other | 4 | 0 |
| **Institutions Represented** |  |  |  |
|  | University of Florida | 36 | 13 |
|  | Emory University | 5 | 0 |
|  | Mayo Clinic | 5 | 0 |
|  | Marshall University | 1 | 0 |
|  | Massachusetts General Brigham | 1 | 0 |
|  | University of Colorado | 0 | 2 |
|  | Benjamin Franklin Cummings Institute of Technology | 1 | 0 |
|  | University of Alabama Birmingham | 1 | 0 |
|  | University of Michigan | 0 | 1 |
|  | University of South Florida | 0 | 1 |
|  | University of California Los Angeles | 2 | 0 |
|  | Brown University | 1 | 0 |
|  | Rutgers University | 1 | 0 |
|  | Yale | 0 | 1 |
|  | Not affiliated with a US academic institution | 9 | 0 |
| **Background** |  |  |  |
|  | Clinical | 37 | 13 |
|  | Data Science/Engineering | 26 | 5 |

Supplementary Material 4.2: Workshop Registrant Demographics by Track
