# Supplementary material for: Building AI competence in the healthcare workforce with the AI for clinical care workshop: A Bridge2AI for clinical CHoRUS project
Source: J Clin Transl Sci. 2025 Oct 3;9(1):e243. doi: 10.1017/cts.2025.10156 (PMC12695489; doi:10.1017/cts.2025.10156)
Supplement: Davidson et al. supplementary material 5 — Davidson et al. supplementary material [file S2059866125101568sup005.docx]

**Building AI Competence in the Healthcare Workforce with the AI for Clinical Care Workshop: a Bridge2AI for Clinical CHoRUS Project**

Andrea E. Davidson, BS, Aiden Jose, Benjamin Shickel, PhD, Kaleb E. Smith, PhD, Parisa Rashidi, PhD, Yulia Levites Strekalova, PhD, MBA, Azra Bihorac, MD, MS

Supplementary Materials 5: Bridge2AI CHoRUS Consortia List as of October 15^th^, 2024

| **Author's name** | **Affiliated Institution** |
| --- | --- |
| Gari D. Clifford | Emory University and Georgia Institute of Technology |
| Eric S. Rosenthal | Department of Neurology, Massachusetts General Hospital, Boston, MA 02114 |
| Sachin Mehta | Department of Anesthesiology, Duke University School of Medicine |
| Michael J. Young, MD, MPhil | Center for Neurotechnology and Neurorecovery, Department of Neurology, Massachusetts General Hospital and Harvard Medical School |
| Mihai V Podgoreanu, MD | Division of Cardiothoracic Anesthesiology and Critical Care, Duke University, Durham, NC |
| Murad Megjhani | 1 Department of Neurology, Columbia University, NY, United States of America 2 Program for Hospital and Intensive Care Informatics, Department of Neurology, Columbia University, NY, United States of America |
| Matthew A. Reyna | Department of Biomedical Informatics, Emory University, Atlanta, GA, USA |
| Craig S. Jabaley, MD, FCCM | (1) Department of Anesthesiology, Emory University School of Medicine, Atlanta, GA, USA; (2) Emory Critical Care Center, Atlanta, GA, USA |
| Joo Heung Yoon | Division of Pulmonary, Allergy, Critical Care, and Sleep Medicine, University of Pittsburgh, Pittsburgh, PA |
| Tom Pollard | MIT Laboratory for Computational Physiology, Institute for Medical Engineering and Science, Massachusetts Institute of Technology, Cambridge, MA 02142 |
| Vitaly Herasevich, MD, PhD | Department of Anesthesiology and Perioperative Medicine, Mayo Clinic, Rochester, MN |
| Andrew E Williams | Tufts Medical Center, Institute for Clinical Research and Health Policy Studies |
| Soojin Park | Columbia University Vagelos College of Physicians and Surgeons, New York-Presbyterian Hospital |
| Jennifer A. Muszynski | Division of Critical Care Medicine, Nationwide Children's Hospital, Columbus, Ohio |
| Xiang Li | Massachusetts General Hospital |
| Paul Vespa | David Gefffen School of Medicine at UCLA |
| Gloria Hyunjung, Kwak, PhD | Center for Data Science, Nell Hodgson Woodruff School of Nursing, Emory University, Atlanta, GA, USA |
| Cynthia Rudin | Duke University |
| Jared Houghtaling | Tufts Medicine - Institute for Clinical Research and Health Policy Studies (ICRHPS) |
